# Professor A. Venugopal: Founder of Urology at Madras Medical College

**DOI:** 10.4103/0970-1591.45530

**Published:** 2009

**Authors:** C. Chinnaswami

**Affiliations:** Madras Institute of Urology, 180, NSK Salai, Chennai – 600 026, India E-mail - drccuro@gmail.com

**Figure:**
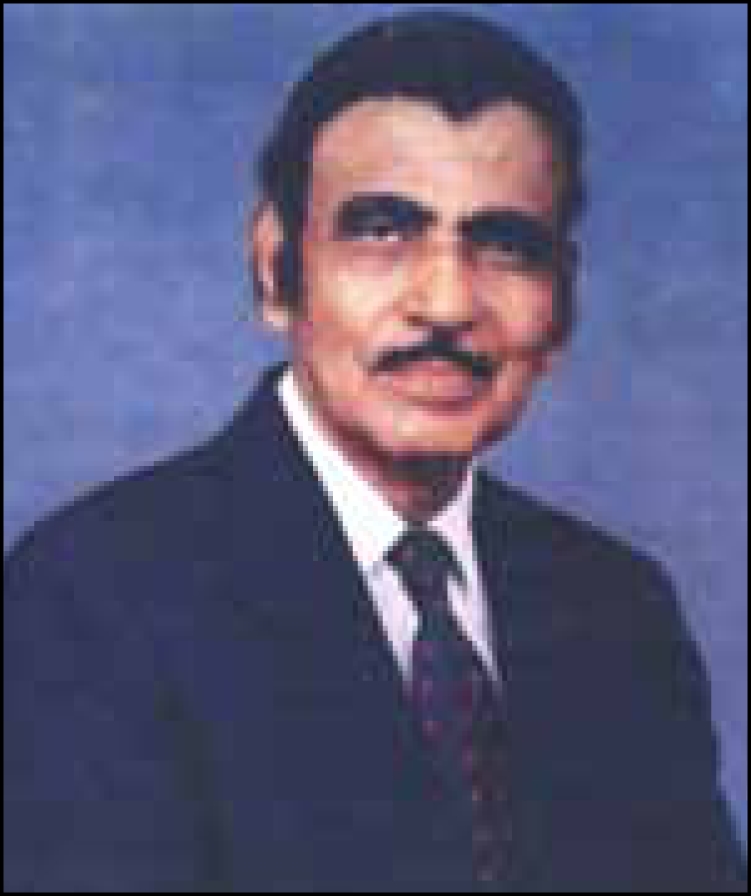
Prof. A. Venugopal

Professor. Arcot Venugopal great pioneer Urologist, an excellent surgeon, fine orator and great teacher was born on 18^th^ June 1917 at Madras. He is the eldest son of great Doyen of Education and Medical Profession, Sir. A. Lakshmanaswami Mudaliar. He graduated in Medicine 1945 both from Madras Medical College in the year 1940 and obtained his Post-Graduate degree in surgery in 1945 both from Madras Medical College.

He had worked under many distinguished surgeons in Madras; he had urological training under Prof. Reed Nesbit, a pioneer urologist in USA at AnnArbor, Michigan.

He was appointed as Honorary Clinical Professor of surgery and Honorary Surgeon, Madras Medical College and Government General Hospital, Madras in the year 1952. This Honorary appointment continued until his retirement as Honorary Professor of Urology in the year 1976. When the Urology Department was started in Madras Medical College in the year 1967, he was designated as Honorary Professor and Head of Department of Urology.

As an Honorary Professor he used to spend more time in the hospital than many of the full time teachers either doing surgery or teaching undergraduate or post-graduate students, truly service beyond the call of duty. He used to bring his own Endoscopes to the General Hospital to operate on public. He had set very high standards of Ethics in practice. His bed side clinics and class room lectures used to attract students from all the medical colleges in the city. Dr. B.N. Colabawala had this to say about his lectures, “As a man of learning he was well versed with general literature and could quote ad-lib, he garnished his talks with such quotes to the delight of many”.

His interest in Medical education and teaching did not end with his retirement as Honorary Professor of Urology. His activities multiplied manifold, he became Honorary Director of Post graduate Institute of Basic medical sciences of the University of Madras, this Institute is now known as A.L. Mudaliar Institute of Basic Medical Sciences and has trained many post graduates doing research activities in Genetics Biochemistry, Microbiology, Pathology, Anatomy etc.,

It is amazing that he was able to spend so much time and energy in academic Committees in spite of his heavy commitment to clinical Urology. He had been a Member or Chairman of numerous committees connected with medical education, hospital administration and other similar bodies. I will mention some of the important Committees with which he was associated.

He was Chairman of the Committee which drafted the proposals for University of Health Sciences which formed the basis for the formation of Medical University in Tamil Nadu and Andhra Pradesh.

He served on the Syndicate Senate, Academic Councils and Board of studies in Medicine in various Universities in the country.

He had been a member of Medical Council of India and member of its Executive Committee from 1975 to 1983.

He had been an Inspector of Medical Council of India and inspected various Medical Colleges and Hospitals in the country and has advised methods to improve various institutions. He has served as Member of the Planning Commission, and Hospital Improvement Committee of Tamil Nadu and was responsible for improving Health Services in the state.

He has delivered many post graduate lectures and Orations in many medical colleges and universities, notable among them are: Yellapragada Subba Rao Oration at Kurnool: Kutumbiah Endowment lectures at Vishakapatnam, Shantilalsheth Oration at Bombay University.

He has many publications to his credit, he was the author of “Text-book on preoperative and post operative care of Surgical patients”, he has contributed chapters in “Text Book of Surgery” edited by Lal and Menon” Text Book of Obstetrics and Gynaecology” by Dr. Sir. A.L. Mudaliar and Dr. M.K.K. Menon. In the “Text Book of Tuberculosis” edited by Dr. K.N. Rao he contributed a chapter on Genito-Urinary tuberculosis.

His service to the Association of Surgeons of India, parent body of Urological Society of India is unique. He had been the Secretary of the body for 22 years, elected unanimously for eight consecutive terms from 1953 to 1975, comparable to the feat of his illustrious father Sir. A.L. Mudaliar who was Vice-Chancellor of Madras University for more than 25 years without a break.

Prof. Venugopal was largely responsible in constructing Headquarters building of the Association of Surgeons of India, at Chennai. He became President of Association of Surgeons of India in the year 1978.

He was president of Urological Society of India in the years 1977 and 1978. It was indeed unique in the history of these associations for a person to hold the post of President of both the associations at the same time.

About Prof. A,V's administrative abilities Dr. B.N. Colabawalla said “he admired his handling of tricky issues even when under fire”.

He was awarded Fellowships of American College of Surgeons in 1955, International College of surgeons in 1956 and Indian Academy of Medical sciences in 1967. Tamil Nadu Dr. M.G.R. Medical University awarded the degree of Doctor of Science-D.Sc in the year 1993 for rendering meritorious services to Medical Education. National Academy Medical Sciences conferred Life time achievement award in the year 2006.

Urology society and Urologists should be greatly indebted to Prof. Venugopal for the services rendered Prof. H.S. Bhat said “Prof. Venugopal prevailed on his great father Sir. A.L. Mudaliar in his persuasive manner, the need for starting a post-graduate course in urology and this is one single factor responsible for the growth of urology as a specialty to its present state”. Dr. Colabawala also said “It was at his instance the first M.Ch (Urology) was established at the University of Madras, which acted as a Catalyst for such courses all over the country”.

As a person, Prof. Bhat said, “I can only describe Prof. Venugopal in one sentence - a gentleman in private and public”.

Today, if Urologists in this country and the Urology society of India commands respect and admiration of the public and academicians it is because of services of persons like Prof. A. Venugopal.

Prof. Venugopal's has two sons Dr. Jayagopal urologist and Dr. Harendra, General Surgeon and a daughter Brinda a biochemist. Mrs. Mohana Venugopal noble lady, kind hearted person is truly the strength behind this great personality. All the residents and assistants who had worked under him will remember how patiently she used to wait everyday in the car as the Honorable surgeon made his routine night rounds in the General Hospital and talking to the residents. Let us pray god almighty to give him strength to give more of active service to the humanity.

